# Long noncoding RNA *CRNDE* stabilized by hnRNPUL2 accelerates cell proliferation and migration in colorectal carcinoma via activating Ras/MAPK signaling pathways

**DOI:** 10.1038/cddis.2017.258

**Published:** 2017-06-08

**Authors:** Huijuan Jiang, Yiqing Wang, Meiling Ai, Haowei Wang, Zhijiao Duan, Huanan Wang, Li Zhao, Jiang Yu, Yanqing Ding, Shuang Wang

**Affiliations:** 1 Department of Pathology, Southern Medical University, Nanfang Hospital, Guangzhou, China; 2 Department of Pathology, School of Basic Medical Sciences, Southern Medical University, Guangzhou, China; 3 Department of General Surgery, Nanfang Hospital, Southern Medical University, Guangzhou, China

## Abstract

Recent studies have furthered our understanding of the function of long noncoding RNAs (lncRNAs) in numerous biological processes, including cancer. This study investigated the expression of a novel lncRNA, colorectal neoplasia differentially expressed (*CRNDE*), in colorectal carcinoma (CRC) tissues and cells by real-time RT-PCR and *in situ* hybridization, and its biological function using a series of *in vitro* and *in vivo* experiments to determine its potential as a prognostic marker and therapeutic target. *CRNDE* was found to be upregulated in primary CRC tissues and cells (*P*<0.05), and the upregulation of *CRNDE* expression is a powerful predictor of advanced TNM stage (*P*<0.05) and poor prognosis for CRC patients (*P*=0.002). The promoting effects of *CRNDE* on the cell proliferation, cell cycling and metastasis of CRC cells were confirmed both *in vitro* and *in vivo* by gain-of-function and loss-of-function experiments. Mechanistically, it was demonstrated that *CRNDE* could form a functional complex with heterogeneous nuclear ribonucleoprotein U-like 2 protein (hnRNPUL2) and direct the transport of hnRNPUL2 between the nucleus and cytoplasm. hnRNPUL2 that was accumulated in the cytoplasm could interact with *CRNDE* both physically and functionally, increasing the stability of *CRNDE* RNA. Moreover, gene expression profile data showed that *CRNDE* depletion in cells downregulated a series of genes involved in the Ras/mitogen-activated protein kinase signaling pathways. Collectively, these findings provide novel insights into the function and mechanism of lncRNA *CRNDE* in the pathogenesis of CRC and highlight its potential as a therapeutic target for CRC intervention.

Colorectal carcinoma (CRC) is the third leading cause of cancer-related deaths worldwide, and its incidence is on the rise.^1^ Although it is well known that multiple known carcinogens and varying genetic backgrounds are involved in the tumorigenesis and progression of CRC, the detailed interactions and regulatory mechanisms of key pathways implicated in the progression of the disease are still obscure. Therefore, there is an urgent need for the identification of a reliable molecule that is involved in the progression of CRC and of novel targets for effective intervention.

Recent improvements have revealed that a substantial portion of the human genome can be transcribed into many short or long noncoding RNAs (lncRNAs).^2, 3^ LncRNAs are transcripts longer than 200 bp that do not have any apparent protein-coding ability. Although to data, only a small number of functional lncRNAs have been well characterized, accumulating data suggest that lncRNAs are powerful transcriptional and post-transcriptional regulators to modulate downstream target genes and participate in diverse physiological and pathological processes.^4^ The aberrant expression of lncRNAs has been demonstrated in multiple malignancies,^5, 6, 7, 8, 9, 10^ including CRC, providing new insights into the pathogenesis of cancer. However, the potential role of lncRNAs in CRC pathogenesis and progression remains obscure.

The novel lncRNA, colorectal neoplasia differentially expressed (*CRNDE*), was originally discovered as an upregulated gene in colorectal adenomas and cancers, whereas there is little to no expression in normal colon epithelia.^11^ At least 10 splice variants of *CRNDE* have been identified. The expression level of *CRNDE-h* transcript was upregulated in the plasma of CRC patients, and the expression levels of this transcript alone have shown a sensitivity of 87% and specificity of 93% for predicting the presence of CRC.^11^ In addition to CRC, *CRNDE* overexpression has been observed in many other solid tumors and lymphocytic leukemias.^12^ In glioma, *CRNDE* is the most highly expressed lncRNA,^12, 13^ and it has been shown to affect the malignant biological characteristics of glioma stem cells.^14^ In ovarian cancer, elevated levels of *CRNDE* were found to be a negative prognostic factor, increasing the risk of death and recurrence in ovarian cancer patients treated with platinum compounds and taxanes.^15^ Very recently, a study has shown that increased expression of *CRNDE* is correlated with a poor prognosis in CRC.^16^ Collectively, the current evidence suggests that *CRNDE* overexpression appears to have a key role in tumorigenesis. However, the precise function and mechanism behind *CRNDE* overexpression in CRC and the downstream molecules associated with its action are largely unknown.

In this study, we demonstrated that increased *CRNDE* expression is a characteristic molecular change in CRC and investigated the effects of aberrant *CRNDE* expression on the cellular biological behavior of CRC cells. We further present evidence of *CRNDE* interacting with heterogeneous nuclear ribonucleoprotein U-like 2 protein (hnRNPUL2) protein and activating Ras/mitogen-activated protein kinase (MAPK) signaling pathways in CRC cells. Our findings provide novel insights into the function and mechanisms of *CRNDE* in CRC pathogenesis and a potential therapeutic target for CRC intervention.

## Results

### *CRNDE* is upregulated in CRC tissues and cell lines

**Figure 1:**
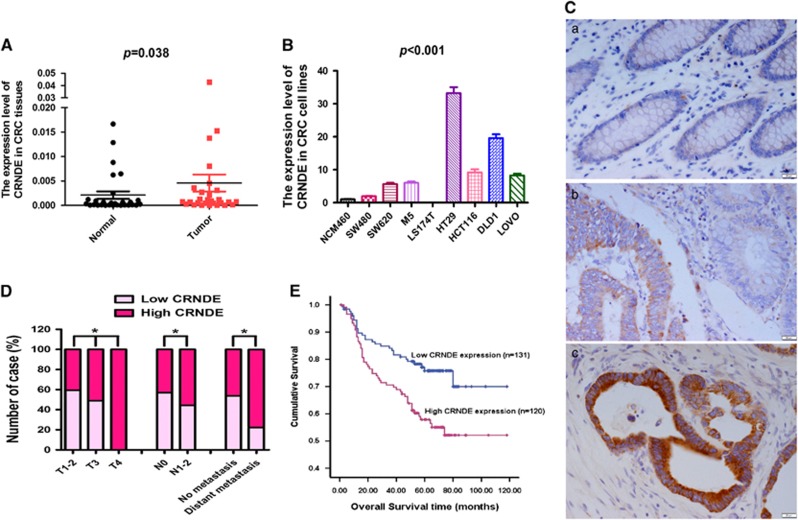
*CRNDE* expression is upregulated in CRC and could be an independent prognostic factor for the prediction of the overall survival of CRC patients. (**A**) Expression levels of *CRNDE* in paired CRC and adjacent non-cancerous tissues. (**B**) Expression levels of *CRNDE* in CRC and colon mucosa epithelial (NCM460) cell lines. (**C**) Expression analysis of *CRNDE* in normal colorectal mucosa and CRC tissues by ISH. (a) Negative expression of *CRNDE* in normal colorectal mucosa. (b) High expression of *CRNDE* in a tumor tissue sample and weak expression of *CRNDE* in its normal mucosal counterpart were observed in one filed of a tissue sample from a single patient. (c) High expression of *CRNDE* in CRC tissue. Scale bars are shown in the lower right corner of each picture. (d) Graphical illustration of statistical *CRNDE* distribution in CRC patients. (**E**) Kaplan–Meier analysis of overall survival in all patients with CRC according to *CRNDE* expression. *P<0.05

The expression levels of *CRNDE* were detected by real-time RT-PCR in a panel of CRC cell lines and 30 paired CRC and adjacent non-cancerous mucosa tissues. An increase in *CRNDE* expression was seen in CRC tissues compared with paired non-cancerous tissues (*P*=0.038, Figure 1A). Compared with a colon mucosa epithelial cell line (NCM460), *CRNDE* expression was higher in seven out of eight CRC cell lines, the exception being LS174T (Figure 1B).

### High *CRNDE* expression is associated with poor prognosis in CRC patients

We assessed *CRNDE* expression in CRC tissues by *in situ* hybridization (ISH). We observed that *CRNDE* was mainly expressed in the cytoplasm and that it was expressed in 89.64% (225 of 251) of all CRC samples, but in only 19.53% (25 of 128) of adjacent non-cancerous tissues. The levels of *CRNDE* were significantly upregulated in CRC tissues (*P*<0.001, Figure 1C). From the clinical data, it was found that high levels of *CRNDE* expression were associated with T stage, N stage and M stage (Figure 1D and Table 1). Furthermore, Kaplan–Meier analysis revealed that higher *CRNDE* expression in CRC tissues was significantly correlated with reduced overall survival in CRC patients (*P*=0.002, log-rank test=7.294; Figure 1E). In addition, multivariate survival analysis was performed including the parameters of gender, age, tumor size, tumor differentiation, T stage, N stage, M stage and *CRNDE* expression level. From this analysis, we identified *CRNDE* expression level as an independent prognostic factor of disease outcome in CRC patients (Table 2).

### Knockdown of *CRNDE* inhibits CRC cell proliferation, invasion and migration *in vitro*

**Figure 2:**
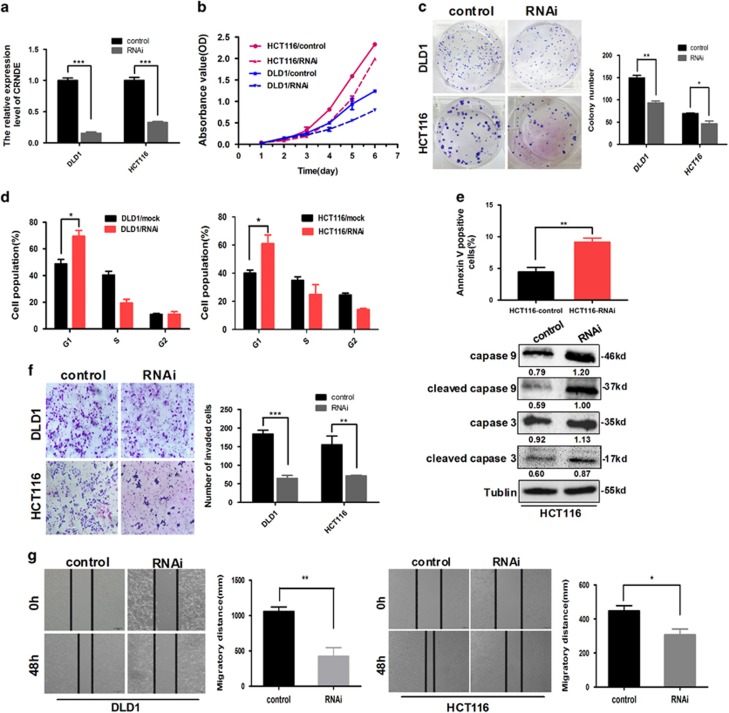
*CRNDE* depletion inhibits cell proliferation, cell cycling, invasion and migration in CRC cells *in vitro*. (**a**) DLD1 and HCT116 cells were infected with Lv-si*CRNDE* to establish two cell lines with stable knockdown of *CRNDE* expression. *CRNDE* levels in DLD1 and HCT116 cells after shRNA-mediated knockdown of *CRNDE* were detected by real-time RT-PCR. (**b**) CCK-8 assays were performed to determine the proliferation of *CRNDE*-depleted CRC cells. (**c**) Colony-forming assays were performed to determine the effects of *CRNDE* depletion on the growth of CRC cells. (**d**) Cell cycle progression was analyzed by flow cytometry. (**e**) Apoptosis assays were performed to determine the effects of *CRNDE* depletion on CRC cells by flow cytometry (upper) and by western blot analysis (lower). (**f**) Matrigel invasion assays were used to determine the effects of *CRNDE* depletion on the invasion ability of CRC cells. (**g**) Scratch-wound-healing assays were performed to determine the effects of *CRNDE* depletion on the migration ability of CRC cells. The experiments were performed in triplicate, and the data are expressed as the mean±S.D. *P<0.05, **P<0.01 and ***P<0.001

To explore the role of *CRNDE* in CRC oncogenesis, we first downregulated *CRNDE* expression by cloning *CRNDE* short-hairpin RNA (shRNA) into lentivirus to establish loss-of-function models in CRC cell lines including in DLD1 and HCT116 cells. Real-time RT-PCR analysis revealed that *CRNDE* was strikingly downregulated in the RNAi group compared with the control group (*P*<0.001, Figure 2a). To clarify whether there are off-target binding sites, we analyzed the knockdown efficiency of *CRNDE* shRNA in CRC cells with ectopic overexpression of *CRNDE*. *CRNDE* levels were significantly reduced again after the *CRNDE*-overexpressing CRC cells were transfected with anti-*CRNDE* siRNAs compared with the control group (Supplementary Figure S1). The Cell Counting Kit-8 (CCK-8) assay was used to explore the biological effects of *CRNDE* on cell proliferation and revealed that the downregulation of *CRNDE* could markedly inhibit CRC cell growth *in vitro* (*P*=0.001, Figure 2b). Similarly, the capacity to form colonies in the *CRNDE*-depleted cells was suppressed compared with the control group (DLD1: *P*=0.002 and HCT116: *P*=0.022, Figure 2c). Moreover, cell cycle progression and apoptosis detection were performed by flow cytometry, which revealed that the depletion of *CRNDE* could result in an increased percentage of G1-phase cells in two of the CRC cell lines (DLD1: *P*=0.020 and HCT116: *P*=0.018, Figure 2d). It was observed that the decrease of *CRNDE* expression could induce cell apoptosis and enhance cleavage/activation of caspase-3 and -9 in HCT116 cells (*P*=0.007, Figure 2e). However, the proportion of apoptotic cells remained similar between DLD1 with *CRNDE*-knockdown and control cells (*P*=0.394, Supplementary Figure S2).

Moreover, we observed the effect of *CRNDE* on the invasion and migration capacity of CRC cells via a Matrigel invasion assay and wound-healing assay. Matrigel invasion analysis demonstrated that the knockdown of *CRNDE* in CRC cells strongly reduced cell invasiveness (DLD1: *P*<0.001 and HCT116: *P*=0.007, Figure 2f). The wound-healing assay also illustrated that downregulation of *CRNDE* expression reduced the cell migration capacity (DLD1: *P=*0.0097 and HCT116: *P=*0.030, Figure 2g). These data suggest that *CRNDE* is necessary for cell invasion and migration.

### Knockdown of *CRNDE* reduces human CRC cell proliferation and metastasis in mice *in vivo*

**Figure 3:**
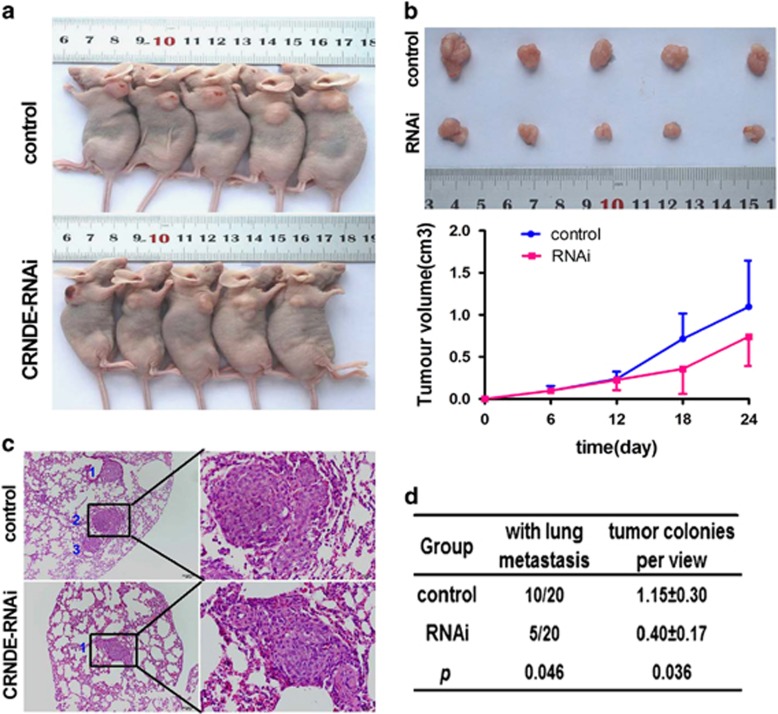
The *CRNDE* depletion inhibits the growth and metastasis of human CRC cells in mice *in vivo*. (**a**) DLD1 cells with stable *CRNDE* downregulation and control cells were inoculated into nude mice. These graphs show the tumor xenografts 4 weeks after ectopic-subcutaneous implantation in nude mice. (**b**) DLD1 cells with downregulated CRNDE expression exhibited attenuated tumor growth in nude mice. The effect of *CRNDE* on CRC tumor growth was evaluated based on tumor volume in the two groups. (**c**) Representative pictures of lung metastasis by hematoxylin and eosin (H&E) staining in nude mice 4 weeks after tail vein injection with *CRNDE* downregulated DLD1 cells or control cells. (**d**) Statistical comparisons of lung metastasis and pulmonary tumor colonies per view in the two groups of mice after tail vein injection

We subcutaneously injected CRC cells with stable *CRNDE* downregulation or control cells into nude mice for xenoplantation. Consistent with the *in vitro* results, mice injected with *CRNDE*-depleted DLD1 cells showed significantly decreased tumor growth compared with those injected with control cells (Figures 3a and b).

Next, we established a peripheral intravascular implanted metastatic model by injecting DLD1 cells with downregulated *CRNDE* expression or negative control cells into nude mice through the tail vein. At 4 weeks after injection, the mice were killed, and the lungs were subjected to histological analysis. The results showed that *CRNDE* knockdown decreased the number of definite pulmonary colonization sites (5 out of 20 mice) compared with the control group (10 out of 20 mice) (*P*=0.046, Figures 3c and d). Moreover, compared with mice injected with control cells, the number of pulmonary tumor colonies per microscopic view in the *CRNDE*-knockdown cell-injected mice was significantly decreased (*P*=0.036, Figure 3d). Taken together, these data indicate an important role for *CRNDE* in promoting tumor metastasis *in vivo*, which is supported by in *vitro* results.

### Overexpression of *CRNDE* promotes CRC cell growth, invasion and migration *in vitro*

**Figure 4:**
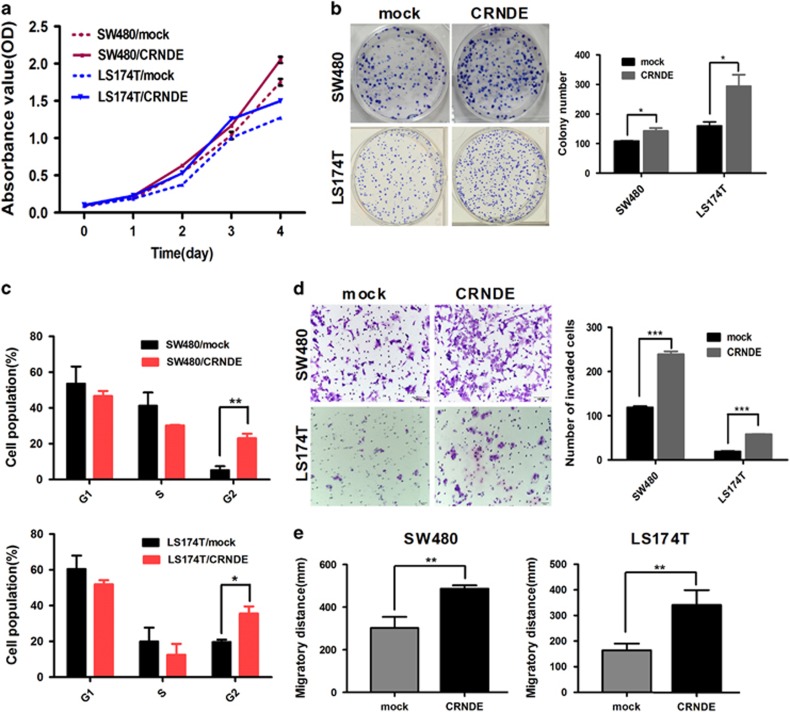
Overexpression of *CRNDE* promotes cell proliferation, cell cycling, invasion and migration in CRC cells *in vitro*. (**a**) Overexpression of *CRNDE* induced significantly higher growth rates in CRC cells. (**b**) Overexpression of *CRNDE* increased the capacity to form colonies in CRC cells. (**c**) Overexpression of *CRNDE* induced a significant increase in cells at G2-phase relative to mock cells. (**d** and **e**) Invasion/migration assays using Matrigel Transwell and wound-healing assays for CRC cells. *CRNDE* overexpression promoted the invasion and migration of CRC cells. The experiments were performed in triplicate; the data are expressed as the mean±S.D. *P<0.05, **P<0.01 and *** *P*<0.001

We also established gain-of-function models to investigate the changes in the biological behavior of SW480 and LS174T cells following *CRNDE* overexpression. Real-time RT-PCR analysis revealed that *CRNDE* was significantly upregulated in both SW480 and LS174T cells that infected with lentivirus carrying the *CRNDE-h* gene compared with the control group (Supplementary Figure S1). As shown in Figure 4a, the CCK-8 assay indicated when *CRNDE* expression was enhancing, the proliferation rate of CRC cells was significantly increased, compared with the mock cells (*P*<0.001). Similarly, compared with the control cell lines, the capacity to form colonies was increased in the *CRNDE*-overexpressing cells (SW480: *P*=0.027 and LS174T: *P*=0.031, Figure 4b). Flow cytometry also revealed that the overexpression of *CRNDE* increased the proportion of G2-phase cells (SW480: *P=*0.006 and LS174T: *P*=0.018, Figure 4c). However, the proportion of apoptotic cells remained similar between *CRNDE*-overexpressing and control cells (*P*>0.05, Supplementary Figure S2). Meanwhile, we also observed that ectopic overexpression of *CRNDE* increased the capacity of invasion (*P*<0.001, Figure 4d) and migration in both SW480 and LS174T cells (SW480: *P=*0.004 and LS174T: *P*=0.001, Figure 4e).

### *CRNDE* binds to hnRNPUL2 protein and directs its localization

**Figure 5:**
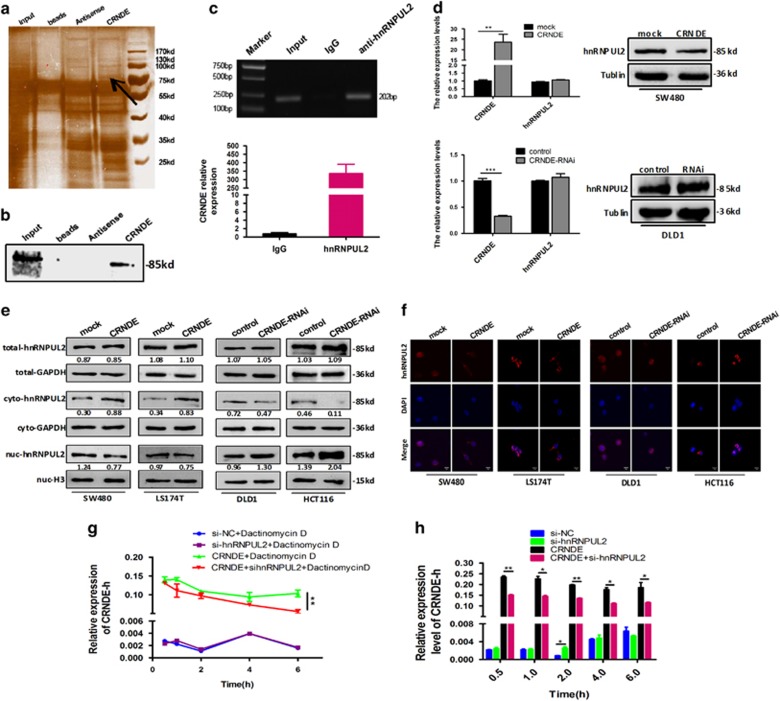
*CRNDE* interacts with hnRNPUL2 protein and directs its localization. (**a**) RNA pull-down assays were used to identify proteins associated with *CRNDE*. Biotinylated *CRNDE* and antisense RNA were incubated with cell extracts, and the associated proteins were resolved by SDS-PAGE. Specific bands were excised and submitted for mass spectrometry, and hnRNPUL2 was identified. (**b**) Western blotting analysis of the specific interaction of hnRNPUL2 with *CRNDE*. (**c**) RIP experiments were performed in DLD1 cells using an hnRNPUL2 antibody or nonspecific IgG, and specific primers were used to detect *CRNDE*. RIP enrichment was determined as the amount of RNA associated with hnRNPUL2 or IgG relative to the input control. (**d**) *CRNDE* did not induce the expression level of hnRNPUL2 in SW480 and DLD1 CRC cells. *CRNDE*-induced subcellular relocalization of hnRNPUL2 protein was identified by western blot analysis (**e**) and immunofluorescence microscopy analysis (**f**). Scale bars=10 *μ*m. Increased hnRNPUL2 was seen in the cytoplasm following *CRNDE* overexpression in SW480 and LS174T cells. Conversely, the downregulation of *CRNDE* could reduce the cytoplasmic levels of hnRNPUL2 expression in DLD1 and HCT116 cells. (**g**) Cytoplasmic hnRNPUL2-stabilized *CRNDE* RNA. Cells were transfected with hnRNPUL2-siRNA or control siRNA for 48 h and were then exposed to actinomycin D (1 *μ*g/ml), and total RNA was isolated at the indicated times and subjected to real-time RT-PCR to assess the half-life of *CRNDE* RNA. (**h**) Cytoplasmic hnRNPUL2 induced *CRNDE* RNA levels in CRC cells

To identify the proteins that bind to *CRNDE*, we performed an RNA pull-down experiment in DLD1 cells. We separated the RNA-associated proteins by sodium dodecyl sulfate-polyacrylamide gel electrophoresis (SDS-PAGE), excised the bands specific to *CRNDE* and subjected them to mass spectrometry (Figure 5a). Interestingly, hnRNPUL2 was the most abundant protein among all of the proteins identified by mass spectrometry (Table 3), suggesting an interaction between hnRUNPUL2 and *CRNDE*. Western blotting analysis also was carried out to detect hnRNPUL2 (Figure 5b). RNA immunopreciptiation (RIP) assay with an antibody against hnRNPUL2 was performed to further validate the interaction between *CRNDE* and hnRNPUL2. Significant *CRNDE* RNA enrichment was observed using the hnRNPUL2 antibody compared with using a nonspecific antibody (IgG control) (*P=*0.004, Figure 5c). These analyses confirmed that *CRNDE* physically associates with hnRNPUL2 *in vitro*.

Furthermore, we measured and compared the levels of hnRNPUL2 expression in CRC cells with *CRNDE* overexpression or *CRNDE* depletion. However, there was no significant difference in either hnRNPUL2 mRNA or total protein levels in SW480 cells with *CRNDE* upregulation or DLD1 cells with *CRNDE* downregulation, compared with that in the control cells (Figure 5d). Although all of the hnRNP proteins are present in the nucleus, some seem to shuttle between the nucleus and the cytoplasm.^17^ It has been reported that lncRNAs can direct the localization of target proteins within cellular compartments. Therefore, we speculated that *CRNDE* might influence the localization of hnRNPUL2 in CRC cells. To clarify our hypothesis, nuclear and cytoplasmic protein fractions were prepared from CRC cells. It was found that the overexpression of *CRNDE* induced a subcellular relocalization of hnRNPUL2 and increased the amounts of hnRNPUL2 that were seen in the cytoplasm. Conversely, the downregulation of *CRNDE* appeared to reduce the cytoplasmic levels of hnRNPUL2 expression in DLD1 and HCT116 cells (Figures 5e and f). These results suggested that *CRNDE* could bind to hnRNPUL2 protein and direct its transition between the nucleus and the cytoplasm.

### Cytoplasm-accumulated hnRNPUL2 stabilizes *CRNDE* RNA

Given the earlier results demonstrating that hnRNPUL2 proteins shuttle between the nucleus and the cytoplasm, it is also possible that they have cytoplasmic functions. Previous studies have implicated hnRNPs in the regulation of mRNA stability.^18, 19^ Therefore, we examined the effects of cytoplasmic hnRNPUL2 protein on the stability of *CRNDE*. *CRNDE*-overexpressing SW480 cells were first transfected with hnRNPUL2-siRNA or control siRNA and then treated with 1 *μ*g/ml actinomycin D over a 6-h period to block new RNA synthesis. Then, *CRNDE* expression was assessed. As shown in Figures 5g and h, the siRNA-mediated hnRNPUL2 depletion was found to reduce *CRNDE* stability and *CRNDE* expression levels following the cytoplasmic retention of hnRNPUL2 that was induced by *CRNDE* overexpression; meanwhile, this reduction of *CRNDE* levels was not observed in no *CRNDE*-overexpressing cells compared with its corresponding controls, implying that an increased content of cytoplasmic hnRNPUL2 following *CRNDE* overexpression leads to an increase in the *CRNDE* expression level. These results collectively suggest that *CRNDE* overexpression induces hnRNPUL2 to accumulate in the cytoplasm, which allows for its interaction with *CRNDE*, leading to an increase in the stability of *CRNDE* expression. Thus, we concluded that the interaction between *CRNDE* and hnRNPUL2 is not only a physical one but also a functional one.

### *CRNDE* upregulates the expression of Ras/MAPK signaling genes

**Figure 6:**
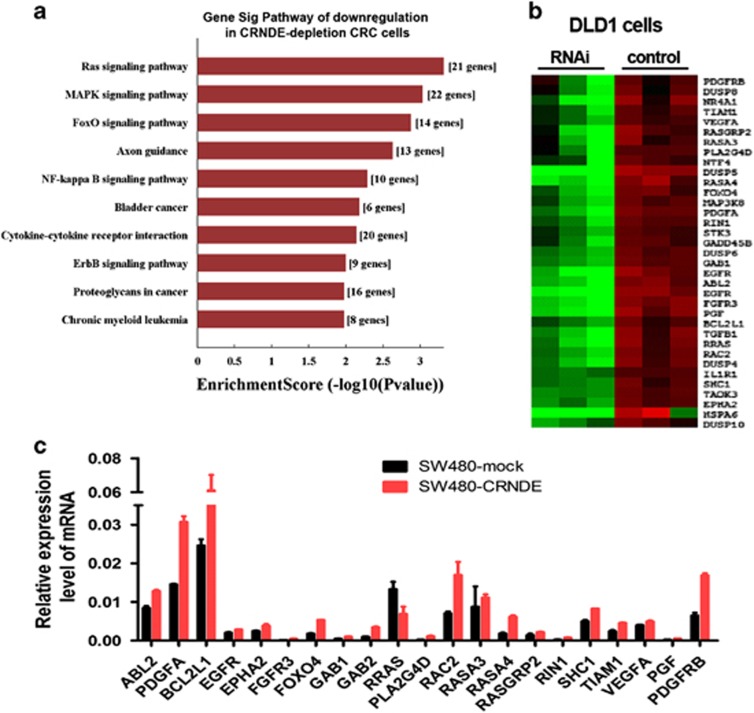
*CRNDE* activates Ras/MAPK signaling pathways. mRNA expression profiles were detected in *CRNDE* knockdown DLD1 cells and control cells. (**a**) KEGG pathway analysis of the genes downregulated in *CRNDE*-depleted cells. (**b**) Hierarchical clustering analysis of Ras/MAPK signaling genes which were downregulated in the *CRNDE*-depleted DLD1 cells. (**c**) A subset of genes was detected by real-time RT-PCR. The experiments were performed in triplicate, and the data are expressed as the mean±S.D.

To identify the genes targeted by *CRNDE* and explore the mechanism of action of *CRNDE* on CRC tumorigenesis, gene expression profiling was performed on *CRNDE*-depleted DLD1 cells and control cells. Many differentially expressed genes (>2-fold; <0.05 false discovery rate) were identified (GSE89985). KEGG pathway analysis indicated that Ras signaling pathway was the main pathway associated with the downregulation of *CRNDE* (Figure 6a). Notably, many pathways known to be associated with cancer, including the MAPK signaling pathway, were found to be deregulated in *CRNDE-*depleted DLD1 cells. The results showed that *CRNDE* affected the expression of a group of functional genes that has similar biological effects in cancer (Figure 6b). Twenty-one Ras signaling genes whose expressions were suppressed in the *CRNDE*-depleted cells were verified in the *CRNDE*-overexpressing CRC cells and control groups by real-time RT-PCR analysis. All genes showed expression trends that were consistent with the gene chip results (Figure 6c).

## Discussion

It is well known that lncRNAs are an important player in cancer biology, typically causing the aberrant expression of gene products that contribute to the progression of a number of human cancers.^7, 20^ A growing body of evidence has indicated that lncRNAs contribute to the progression of CRC. For example, lncRNA *CCAL* regulates CRC progression by activating the Wnt/*β*-catenin signaling pathway via the suppression of activator protein 2*α*. The overexpression of *CCAL* can be used as an indicator of poor survival and can predict the response to adjuvant chemotherapy in CRC patients.^21^ *CRNDE* is a lncRNA that was originally identified in CRC.^11^ However, the function and underlying mechanisms of *CRNDE* in influencing CRC progression are still largely unknown. In the present study, we studied a large cohort of CRC patients and determined that high-expression levels of *CRNDE* were significantly associated with aggressive stages and cancer-related deaths of CRC patients. This association was independent of other clinical covariates, indicating that *CRNDE* expression may be a useful prognostic biomarker to identify patients at a higher risk of CRC progression. While this manuscript was in preparation, Liu *et al.*^16^ used real-time RT-PCR to demonstrate that upregulation of *CRNDE*-*h* was significantly correlated with large tumor size, metastasis and poorer overall survival. This was consistent with our work presented here. Based on the findings of a previous study, which suggested that *CRNDE-h* is expressed at a higher level in the plasma of CRC patients than in that of healthy individuals, we suggest that *CRNDE* may have attractive clinical utility in the screening, diagnosis and prognostication for CRC.

Moreover, we applied *in vitro* and *in vivo* methods to reveal the involvement of *CRNDE* in CRC tumorigenesis, as a tumor promoter and as a facilitator of CRC growth and metastasis. *CRNDE* has been suggested to have an oncogenic role in glioma,^12, 13, 14^ renal cell carcinoma^22^ and gallbladder carcinoma.^23^ Taken together, these data suggest that *CRNDE* has an important role in carcinogenesis, particularly in CRC. Meanwhile, our results confirmed that the knockdown of *CRNDE* by siRNA could reverse the malignant phenotype of CRC. Thus, a potential therapeutic target of *CRNDE* is revealed, suggesting that *CRNDE* depletion may decrease tumor growth and improve survival in CRC patients. Further studies are required to establish the clinical availability of *CRNDE* inhibition as a novel therapeutic strategy.

We investigated the mechanisms of action by which *CRNDE* exerts its modulatory effect on malignant CRC phenotypes. LncRNAs enriched in the cytoplasm typically participate in post-transcriptional regulation by interacting with microRNA or mRNA.^24, 25, 26^ A previous study reported that *CRNDE* decreased the expression of XIAP and PAK7 by binding to and inhibiting miR-186, subsequently affecting the malignant biological characteristics of glioma stem cells.^14^ However, we assume that this is not the primary function of *CRNDE*. LncRNAs have been reported to exert their function by various mechanisms, including interacting with proteins to modulate protein function, regulating protein–protein interactions and directing localization within cellular compartments. These interactions are central to determining the functional effects of lncRNA. An RNA pull-down assay revealed that *CRNDE* could combine with the hnRNPUL2, increasing the cytoplasmic translocation of hnRNPUL2 protein.

As RNA-binding proteins, hnRNPs have been implicated in diverse cellular processes, such as modulating splicing,^27^ mRNA transport,^28^ miRNA maturation^29^ and mRNA stability.^19^ It has been shown that hnRNPUL2 could interact with NBS1, a subunit of the DNA double-strand break (DSB) sensor complex MRE11-RAD50-NBS1, and be recruited to sites of DNA damage to stimulate DNA-end resection and promote DSB repair.^30^ However, the functional roles of hnRNPUL2 remain largely obscure.

The present study identifies hnRNPUL2 as an important player in regulating *CRNDE* expression. The majority of hnRNPUL2 is retained in the nucleus. Only a small fraction of hnRNPUL2 is in the cytoplasm. Our study suggested that *CRNDE* overexpression led to an increase in hnRNPUL2 accumulation in the cytoplasm and that this cytoplasmic hnRNPUL2 was responsible for the interaction with *CRNDE*. Increased cytoplasmic accumulation of hnRNPUL2 was accompanied by increased expression of *CRNDE* as a result of the increased stability of *CRNDE* by hnRNPUL2-bound *CRNDE* in the cytoplasm. Thus, we concluded that there exists a feedback loop between *CRNDE* and hnRNPUL2.

Recent studies have shown that the hnRNP family interacts with multiple lncRNAs. Huarte *et al.*^31^ identified that the p53-inducible lncRNA-p21 bound to hnRNAK to mediate gene repression in response to DNA damage. Similarly, lncRNA *CASC11* has also been reported to interact with hnRNPK and activate the Wnt/*β*-catenin pathway to promote growth and metastasis in CRC.^32^ These findings make it tempt to conclude that hnRNP proteins are widespread mediators of lncRNA function. The present study provides an additional mechanism by which hnRNPUL2 may affect lncRNA stability. Thus, the present findings supplement and extend the current knowledge on the functional role of RNA–protein complexes.

Another major finding of the present study is the identification of downstream molecules associated with *CRNDE* function. The data obtained from the mRNA expression profile of *CRNDE*-depleted CRC cells indicate that *CRNDE* has a broad regulatory function involving the Ras and MAPK pathways. Activation of Ras signaling is arguably the most common biomolecular event in human cancer.^33^ Activated Ras activates a series of kinase cascades, including RAF, MEK and MAPK. These have functions as molecular switches for signaling pathways regulating cell proliferation, survival, migration, differentiation and cytoskeletal dynamism.^34^ However, in reality, many other proteins are also involved in this pathway, including EGFR,^35^ GRB2,^36^ and VEGF and the PDGF family.^37, 38^ A number of researchers have demonstrated that upon stimulation with growth factors, or other inputs, the Ras/MAPK system can promote cell proliferation, differentiation and/or migration.^39, 40^ Of note, many of the Ras/MAPK signaling genes induced by *CRNDE* are known positive regulators of CRC tumorigenesis, including Tiam1,^41, 42^ GRB2,^43, 44^ and RIN1.^45^ These data collectively suggest that *CRNDE* is a widespread mediator of genes involved in the progression of CRC. Future work will be need to elucidate the mechanism by which *CRNDE* regulates the expression of those genes and to further delineate the network controlled by *CRNDE* in CRC progression.

In summary, this study shows that increased *CRNDE* expression is a characteristic molecular change in CRC and that the upregulation of *CRNDE* expression is a powerful predictor of advanced TNM stage and poor prognosis for CRC patients. The elevation of *CRNDE* expression promotes cell proliferation and metastasis, implying that *CRNDE* inhibition can be a novel therapeutic modality for CRC patients. We suggest that cytoplasmic hnRNPUL2 is an important mediator that induces *CRNDE* overexpression via increasing the stability of *CRNDE*, followed by activating the Ras/MAPK signaling pathways. Our findings provide novel insights into the functions and mechanisms of lncRNA *CRNDE* in the pathogenesis of CRC and highlight its potential as a therapeutic target for CRC intervention.

## Materials and Methods

### Ethics statement

The use of tissues for this study has been approved by the ethics committee of Nanfang Hospital, Southern Medical University (Guangzhou, China). All of the patients provided signed, informed consent before the use of these clinical materials for research purposes. This study was carried out in strict accordance with the recommendations in the Guide for the Care and Use of Laboratory Animals of the National Institutes of Health. The protocol was approved by the Committee on the Ethics of Animal Experiments of Southern Medical University.

### Tissue specimens and cell culture

All CRC specimens were obtained from patients who had been diagnosed with primary CRC and had subsequently undergone elective surgery in Nanfang Hospital, Southern Medical University. Freshly frozen tumor samples from 30 CRC patients were selected for real-time RT-PCR. Formalin-fixed tumor tissue samples, comprising of 251 CRC tumor tissues and 128 adjacent non-tumors tissues, were used for ISH analysis. A comprehensive set of clinicopathological data was recorded. Complete follow-up, which ranged from 1 to 117 months, was achieved for all patients, and the median survival duration was 57 months.

The human CRC cell lines DLD1, HCT116, SW480, SW620, LoVo, LS174t and HT29 were obtained from the American Type Culture Collection (ATCC, Manassas, VA, USA). A subclone named M5 with enhanced metastatic abilities in the liver was isolated by the *in vivo* selection of SW480 cells in our laboratory.^46^ All CRC cell lines were cultured in RPMI 1640 medium (Gibco, Gaithersburg, MD, USA) supplemented with 10% fetal bovine serum (Gibco BRL, Gaithersburg, MD, USA) at a humidity of 5% CO_2_ at 37 °C.

### ISH and evaluation of *CRNDE* staining

ISH was performed according to the manufacturer’s protocol (Boster Bio-Engineeting Company, Wuhan, China). Briefly, 4-*μ*m-thick paraffin-embedded sections were deparaffinized with xylene and rehydrated with dilute ethanol of reagent grade. The samples were digested with proteinase K, fixed in 4% paraformaldehyde, hybridized with the 5′-digoxin-labeled probe of *CRNDE* with a sequence of 5′-CCTCAGTTGTCACGCAG- AAG-3′ at 55 °C overnight, and subsequently incubated for 30 min at 4 °C with HRP. Diaminobenzidine was used to develop the stain with a color reaction.

The ISH-stained tissue sections were reviewed and scored separately by two blinded pathologists. Scores were determined using a relatively simple, reproducible scoring method based on both the intensity and proportion of *CRNDE*-positive cells.^46^ The staining intensity was scored on a scale of 0–3, as follows: negative (no staining, 0), weak (1), medium (2) or strong (3). The extent of the staining was defined as the percentage of the positive stained areas of tumor cells or normal epithelial cells in relation to the whole tumor area or the entire section of the normal samples, and it was scored on a scale of 0–4 as follows: 0% (0); 1–25% (1); 26–50% (2); 51–75% (3); and 76–100% (4). The sum of the staining-intensity and staining-extent scores was used as the final staining score for *CRNDE* (on a scale of 0–7). A final staining score of **⩾**3 was considered to denote high-expression of *CRNDE*.

### Construction of cell lines with stably downregulated *CRNDE*

Three shRNA sequences specially targeting *CRNDE* were designed and synthesized (Supplementary Information), and cloned into a pGU6/GFP/Neo-shRNA vector (GenePharma, Shanghai, China). The most effective shRNA sequence in achieving knockdown of *CRNDE* expression was selected for further experiments. A scrambled shRNA oligo, namely, the pGU6/GFP/control, which does not match any known human gene, was used as a control. Stable cell lines of reduced *CRNDE* expression were constructed in DLD1 and HCT116 cells by a fluorescence-activated cell sorting (FACS) analysis for GFP expression as previously described.^47^ Briefly, the cells were resuspended in sterile PBS at a concentration of 1 × 10^7^ cells per ml. GFP-positive cells were identified using a 488-nm argon laser by a BD FACSCanto II Flow Cytometer (BD Bioscience, San Jose, CA, USA). GFP fluorescence was collected through a 530/40 nm bandpass filter. Cells were sorted at a rate of 30 000 events per second using a sort purify 1 drop window. GFP-positive cells were sorted into RPMI 1640 medium supplemented with 10% FBS and plated.

### Construction of CRC cell lines with overexpressed *CRNDE*

To evaluate the expression of *CRNDE*, the *CRNDE-h* sequence was synthesized and subcloned into a pcDNA3.1 vector. CRC cell lines with ectopic expression of *CRNDE* were achieved by pcDNA3.1-*CRNDE* transfection and CRC cells transfected with empty pcDNA vector were used as control (mock). The expression levels of *CRNDE* were detected by real-time RT-PCR.

### RNA isolation and real-time PCR

Total RNA was extracted using TRIzol Reagent (Takara, Dalian, China). RNA fractionation into cytoplasmic and nuclear lysates was carried out using Protein and RNA Isolation System Kit (Ambion by life Technologies, Carlsbad, CA, USA) according to the manufacturer’s instruction. cDNA was synthesized using the PrimeScript RT Reagent Kit (Takara). Real-time RT-PCR was performed to detect the expression of *CRNDE* using One-Step SYBR PrimeScript RT-PCR Kit (Takara). The results were normalized to the expression of *β*-actin. The assay was performed in triplicate for each case to allow for the assessment of technical variability. The primer sequences used for PCR are listed in Supplementary Information.

### Cell proliferation assay and colony formation assay

Cells were seeded in 96-well plates at 2 × 10^3^ cells per well. Cell proliferation was evaluated using CCK-8 (Dojindo, Rockville, MD, USA) according to the manufacturer's instructions. For the colony formation assay, the cells were plated in 6-well plates at 2 × 10^2^ cells per well and maintained in RPMI 1640 containing 10% FBS for 2 weeks. After 2 weeks, the cells were washed two times with PBS, fixed with methanol and stained with Giemsa. The number of colonies was counted under a microscope. All experiments were performed in triplicate.

### Wound-healing and invasion assays

Cell migration was assessed by measuring the movement of cells into a scraped, acellular area that was prepared as described previously.^48^ The spread extent of wound closure was observed after 0 and 48 h, respectively. Migration was quantified by counting the total number of cells that migrated toward the original wound field. For the invasion assay, Matrigel-coated chambers (BD Biosciences, San José, CA, USA) containing 8-*μ*m pores were used. A total of 2 × 10^5^ concentration cells were seeded into the upper chambers (coated in Matrigel) in serum-free medium. The lower chamber of the Transwell was filled with culture media containing 10% FBS as a chemo-attractant. After the chambers were incubated at 37 °C for 48 h, non-invaded cells on the top of the Transwell were scraped off with a cotton swab. Successfully translocated cells were fixed with 10% formalin. Then, they were stained with 0.1% crystal violet for 30 min and counted under a light microscope. All experiments were performed in triplicate.

### *In vivo* functional assays in mouse models

Balb/C-nu/nu nude mice were obtained from the Laboratory Animal Center of Southern Medical University. For *in vivo* tumorigenicity, *CRNDE-*depleted DLD1 cells and control cells were trypsinized, counted and resuspended in sterile PBS. A total of 5 × 10^6^ *CRNDE-*depleted DLD1 cells or control cells were subcutaneously injected into the right and left bilateral upper limbs of mice (4–6 weeks of age), respectively. The mice were then monitored for tumor volume and overall health. The size of the tumor was determined by caliper measurement of the tumor mass. Tumor volume was calculated according to the formula 0.5 × length × wildth^2^. Each experimental group contained five mice.

For developing the *in vivo* metastatic model, mice were injected intravenously via the lateral tail vein with 5 × 10^6^ cells. After 4 weeks of monitoring, mice were killed by cervical dislocation. The lungs were removed by dissection away from adjacent organs, and fixed using 10% neutral-buffered formalin. Subsequently, the consecutive tissue sections were obtained and stained with hematoxylin-eosin (H&E) to observe the metastatic nodules of the lungs under the microscope.

### Western blot analysis

Protein lysates from cells were separated by 10% SDS-PAGE, transferred to 0.22-*μ*m NC membranes (Sigma, Shanghai, China) and incubated with anti-hnRNPUL2 rabbit polyclonal antibody (1: 2000; Abcam, Cambridge, UK) or rabbit polyclonal antibodies against caspase-9, cleaved caspase-9, caspase-3 and cleaved caspase-3 (1:500; CST, Danvers, MA, USA). The band intensity was measured by densitometry using the Quantity One Software (Bio-Rad, West Berkeley, CA, USA). The protein levels were normalized with that of tubulin, GAPDH or histone H3 (1: 1000; Proteintech Group Inc., Wuhan, China). All experiments were repeated in triplicate, and the representative results were shown.

### Immunofluorescence analysis

Different CRC cell lines were cultured and fixed on 12 × 12-mm glass slides. After incubating with antibodies specific for hnRNPUL2 (Abcam) and then with goat anti-rat IgG (Alexa Fluor 594; Invitrogen, Carlsbad, CA, USA), the slides were mounted by adding DAPI-Fluoromount-G (Southern Biotech, Birmingham, AL, USA) and examined with an Olympus FV1000 confocal laser scanning biological microscope (Olympus Corporation, Tokyo, Japan).

### RNA pull-down assays

RNA pull-down assays were performed as described previously.^21^ Briefly, *CRNDE-h* and its antisense RNA were *in vitro* transcribed from the vector pcDNA3.1-*CRNDE*. RNAs were biotin-labeled with the Biotin RNA Labeling Mix (Roche Diagnostics, Indianapolis, IN, USA) and *in vitro* transcribed using the T7/SP6 RNA polymerase (MEGAscript Kits; Ambion). The protein extracted from DLD1 cells was mixed with biotinylated RNAs, incubated with magnetic beads (Life Technologies, Carlsbad, CA, USA) and washed. The retrieved proteins were resolved by SDS-PAGE, and then silver-stained. Specific bands were excised and analyzed by mass spectrometry.

### RNA immunopreciptiation

RIP assays were performed according to the instructions provided in the Magna RIP RNA-Binding Protein Immunoprecipitation Kit (Millipore, Billerica, MA, USA). Briefly, cells were crosslinked with 1% (w/v) formaldehyde and suspended in lysis buffer containing a protease inhibitor cocktail and an RNase inhibitor. Magnetic beads were preincubated with an anti-rabbit IgG or anti-rabbit hnRNPUL2 antibody for 30 min at room temperature, and lysates were then immunoprecipitated with the beads at 4°C overnight. RNA was purified from the RNA–protein complexes that bound to the beads and then was analyzed by real-time RT-PCR.

### Microarray analysis

DLD1 cells following shRNA-mediated knockdown of *CRNDE* and control cells were used for gene expression profiling analysis. Total RNA was extracted from the above-mentioned cell lines, amplified and transcribed into fluorescent cRNA using the Quick Amp Labeling Kit (Agilent Technologies, Palo Alto, CA, USA). The labeled cRNA was then hybridized onto the Human Genome Oligo Microarray (4x44K; Agilent Technologies, Santa Clara, CA, USA), and after the washing steps, the arrays were scanned by the Agilent Scanner G2505C (Agilent Technologies). The Agilent Feature Extraction software (version 11.0.1.1, Agilent Technologies) was used to analyze the acquired array images. Quantize normalization and subsequent data processing were performed using the GeneSpring GX v12.1 software package (Agilent Technologies). The differentially expressed mRNAs with statistical significance were identified using volcano plot filtering. The threshold we used to screen upregulated or downregulated lncRNAs is a fold change⩾2 and a *P*-value⩽0.05. The experiments were performed in triplicate.

### Statistical analysis

All statistical analyses were performed using the SPSS 19.0 software (IBM, Armonk, NY, USA). Differences between groups were identified using a two-tailed Student’s *t*-test. Associations between *CRNDE* expression and clinicopathologic characteristics were determined by the *χ*^2^ test. Survival curves were plotted by the Kaplan–Meier method and compared by the log-rank test. The significance of various variables for survival was analyzed by the Cox proportional hazards model for multivariate analyses. A probability value of 0.05 or less was considered significant.

## Figures and Tables

**Table 1:** Correlation between the clinicopathological features and expression of *CRNDE*

**Characteristics**	***n***	***CRNDE* expression**		
		**Low (%)**	H**igh (%)**	***P*-value**
*Gender*				
Male	150	77 (51.33)	73 (48.67)	0.074
Female	101	54 (53.47)	47 (46.53)	
				
*Age (years)*				
<50	113	66 (58.41)	47 (41.59)	0.74
⩾50	138	65 (47.10)	73 (52.90)	
				
*Tumor size (cm in diameter)*				
<5	100	57 (57.00)	43 (43.00)	0.215
⩾5	151	74 (49.01)	77 (50.99)	
				
*Tumor differentiation*				
Good	96	45 (46.88)	51 (53.12)	0.058
Moderate	99	49 (49.49)	50 (50.51)	
Poor	56	37 (66.07)	19 (33.93)	
				
*T stage*				
1–2	59	35 (59.32)	24 (40.68)	0.027
3	186	96 (51.61)	90 (48.39)	
4	6	0 (0.00)	6 (100.00)	
				
*N stage*				
0	156	89 (57.05)	67 (42.95)	0.048
1–2	95	42 (44.21)	53 (55.79)	
				
*M stage*				
0	240	129 (53.75)	111 (46.25)	0.021
1	11	2 (18.18)	9 (81.82)	

Abbreviations: CRNDE, colorectal neoplasia differentially expressed

**Table 2:** Summary of overall survival analyses by univariate and multivariate Cox regression analysis

**Variables**	**Univariate analysis**			**Multivariate analysis**		
	***P***-**value**	**HR**	**CI (95%)**	***P***-**value**	**HR**	**CI (95%)**
Gender	0.483	1.169	0.756–1.808			
Age	0.686	1.094	0.707–1.693			
Tumor size	0.023	0.576	0.358–0.927	0.003	0.467	0.287–0.767
Tumor differentiation	0.021	1.427	1.055–1.930	0.032	1.443	1.033–2.017
T stage	0.001	2.33	1.391–3.904	0.078	1.565	0.951–2.575
N stage	0.001	2.118	1.372–3.269	0.006	1.885	1.196–2.970
M stage	<0.001	7.451	3.779–14.690	<0.001	6.312	2.935–13.576
*CRNDE* expression	0.002	2.024	1.291–3.173	0.032	1.693	1.047–2.738

Abbreviations: CI, confidence interval; CRNDE, colorectal neoplasia differentially expressed; HR, hazard ratio

**Table 3:** Mass spectrometry analysis of the proteins pulled down by *CRNDE*

**Hits**	**Protein mass**	**No. of peptide**	**Sequence header**	**Relative abundance (%)**
1	130 292.9	7	>sp|P11498|PYC_HUMAN Pyruvate carboxylase, mitochondrial OS=Homo sapiens GN=PC PE=1 SV=2	16.40
2	111 752.4	4	>tr|B4E3S1|B4E3S1_HUMAN cDNA FLJ57040, highly similar to Myosin-9 OS=Homo sapiens PE=2 SV=1	20.00
3	122 461.4	3	>sp|O00159|MYO1C_HUMAN Unconventional myosin-Ic OS=Homo sapiens GN=MYO1C PE=1 SV=4	12.10
4	93 232.38	2	>sp|Q05682|CALD1_HUMAN Caldesmon OS=Homo sapiens GN=CALD1 PE=1 SV=3	8.10
5	85 622.37	2	>sp|Q1KMD3|HNRL2_HUMAN Heterogeneous nuclear ribonucleoprotein U-like protein 2 OS=Homo sapiens GN=HNRNPUL2 PE=1 SV=1	25.90
6	82 434.09	2	>sp|P14923|PLAK_HUMAN Junction plakoglobin OS=Homo sapiens GN=JUP PE=1 SV=3	8.60
7	80 906.33	1	>sp|Q92841|DDX17_HUMAN Probable ATP-dependent RNA helicase DDX17 OS=Homo sapiens GN=DDX17 PE=1 SV=2	8.80

Abbreviation: CRNDE, colorectal neoplasia differentially expressed
