# Supplementary material for: Long noncoding RNA CRNDE stabilized by hnRNPUL2 accelerates cell proliferation and migration in colorectal carcinoma via activating Ras/MAPK signaling pathways
Source: Cell Death Dis. 2017 Jun 8;8(6):e2862–. doi: 10.1038/cddis.2017.258 (PMC5520914; doi:10.1038/cddis.2017.258)
Supplement: Supplementary Information [file cddis2017258x1.docx]

**Long noncoding RNA** ***CRNDE* stabilized by hnRNPUL2 accelerates cell proliferation and migration in colorectal carcinoma via activating Ras/MAPK signaling pathway**


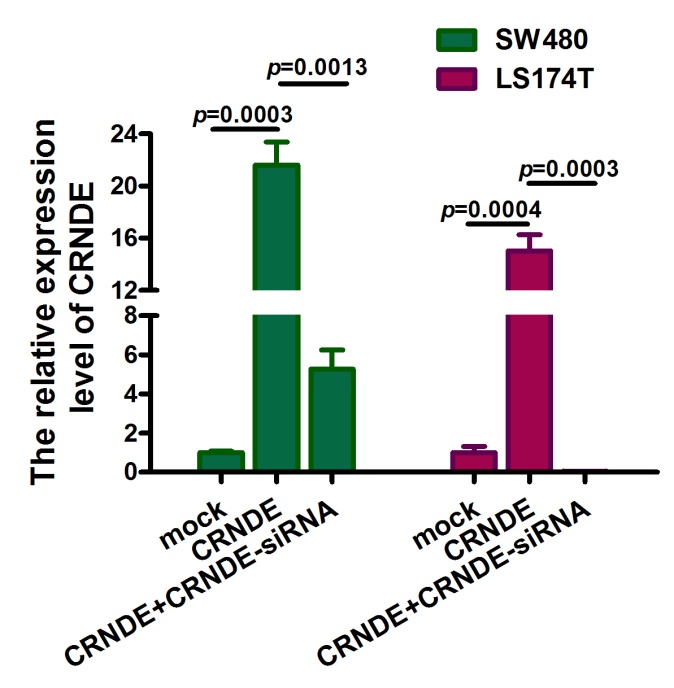


Figure S1. *CRNDE* levels in CRC cells were detected by real-time RT-PCR. Mock demotes CRC cells transfected with empty pcDNA3.1 vector as control. CRNDE demotes CRC cell lines with ectopic expression of *CRNDE* by transfecting pcDNA3.1-*CRNDE*. CRNDE+CRNDE-siRNA demotes that the *CRNDE-*ovexpression CRC cells were knocked down the *CRNDE* expression by shRNA-mediated RNAi.


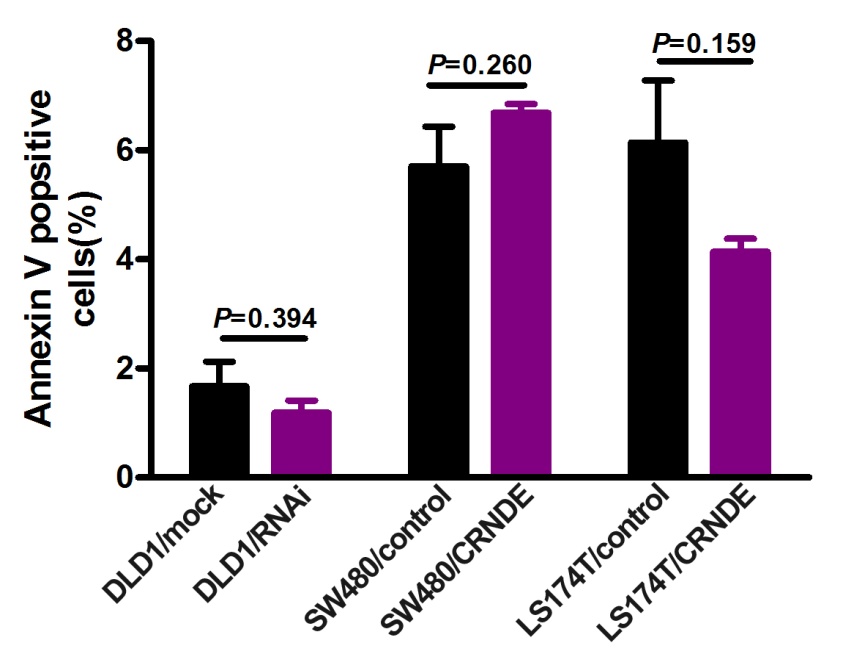


Figure S2. Effects of *CRNDE* on cell apoptosis of CRC cells.

**Supplemental Information**

**Real-time RT-PCR Primer:**

CRNDE-h:

Forward primer 5’-GCGGAGGAGAGGTGTTAAGTGT-3’,

Reverse primer 5’-AACAGGTTTTACCTCCTTATCTTCAGAA-3’.

β-Actin:

Forward primer: 5’-TAAGGAGAAGCTGTGCTACG-3’,

Reverse primer: 5’-GACTCGTCATACTCCTGCTT-3’.

**shRNA sequence of targeting CRNDE-h**

CRNDE-h-homo-783:

sense 5’- GUGCUCGAGUGGUUUAAAUTT-3’,

antisense 5’-AUUUAAACCACUCGAGCACTT-3’;

CRNDE-h-homo-684:

sense 5’-GCCACUGGAAAUGUUGAAATT-3’,

antisense 5’-UUUCAACAUUUCCAGUGGCTT-3’;

CRNDE-h-homo-426:

sense 5’-GUGUGAUGCUUCCAUAAUATT-3’,

antisense 5’-UAUUAUGGAAGCAUCACACTT-3’;

negative control：

sense 5’-UUCUCCGAACGUGUCACGUTT-3’,

antisense 5’-ACGUGACACGUUCGGAGAATT-3’.
